# Migrant Women-experiences from the Mediterranean Region

**DOI:** 10.2174/1745017902016010101

**Published:** 2020-07-30

**Authors:** Caterina La Cascia, Giulia Cossu, Jutta Lindert, Anita Holzinger, Thurayya Zreik, Antonio Ventriglio, Dinesh Bhugra

**Affiliations:** 1 Department of Biomedicine, Neurosciences and advanced Diagnostics, University of Palermo, Palermo, Italy; 2 Department of Medical Sciences and Public Health, University of Cagliari, Cagliari, Italy; 3 Department of Social Work and Health, University of Emden, Emden, Germany; 4 Department for Medical Education, Medical University of Wien, Vienna, Austria; 5 National Mental Health Program, Ministry Of Public Health, Beirut, Lebanon; 6 Department of Clinical and Experimental Medicine, Section of Psychiatry, University of Foggia, Foggia, Italy; 7 Institute of Psychiatry, Psychology and Neuroscience, King’s College London, London, UK

**Keywords:** Women, Migration journey, Violence, Mental health, Mediterranean region, Gender differences

## Abstract

**Introduction:**

The phenomenon of migration is characterized and influenced by a number of different variables; and the different stages of journey are related to different levels and types of psychological distress. Women, in particular, are exposed to further specific risks during migration.

**Aim:**

To determine the factors that affect the psychological health of migrant women during the different stages of the migration journey.

**Methods:**

We provide a narrative review of the literature around the experiences of women during migration process, with a geographical focus on women migrating to the Mediterranean area.

**Results:**

Little data is currently available on the burden of mental health disorders for female migrants. Most studies about the mental health status of migrants were not gender-disaggregated or focused specifically on migrant women’s experiences of violence. Sexual and Gender-Based Violence (SGBV) was found to be a common risk factor faced by all the women who leave their native country to migrate to other countries.

**Conclusion:**

Despite the importance of the issue and the gender-specific variables related to the experience of migrant women, few studies have looked specifically at psychological variables and mental health status in the female migrant population. It is crucial that future studies are conducted around female migration, violence towards women, and women’s mental health, in order to provide an evidence-base for promoting adequate policies and prevention/treatment programs for women.

## 1 INTRODUCTION

Migration is a universal phenomenon that has been ongoing for millennia. However, due to changes in geo-political factors, recent levels of migration to Europe through the Mediterranean region have become of major interest to policymakers and researchers alike. Migration and experiences related to migration are affected by a number of factors, and gender differences are of paramount importance in understanding the stressors experienced by migrants.

## 2 METHODS

A narrative review of the literature was conducted on the phenomenon of migration in the Mediterranean area, and specifically on the psychological and psychosocial experience of migrant women. We searched english-language publications in Pubmed, Scopus, ScienceDirect and PsycInfo. We used the search terms, “women” “migration” “mental health”, “psychological, distress” “mediterranean”, “Sexual and Gender-Based Violence (SGBV)”,“mutilation”, “Intimate Partner Violence Against Women (IPVAW)”, “Female Genital Mutilation (FGM)”, also checking references and bibliography of selected publications to identify additional relevant material.

## 3 RESULTS

### 3.1 Migration in the Mediterranean Area

According to the data from the United Nation High Commission for Refugees (UNHCR) about new arrivals in the European countries, the number of refugees and migrants who arrived by sea to the European coast (Italy, Greece, Spain and Cyprus) after crossing the Mediterranean Sea was 97,546 at the end of October 2018. In previous years, these numbers were considerably greater at 172,301 arrivals in 2017, 362,000 in 2016, and a massive 1,015,078 in 2015. This number is a notable increase from that in 2014, when only 216.054 arrived on the coasts of Europe [1].

Despite the more recent reduction in the number of migrant and refugee arrivals, which could possibly be attributed to changes in the European policies the situation remains challenging from many points of view. People arriving at European coasts are often affected by physical and psychological diseases and illness [2]; either present before the migration from home countries, or caused by conditions of the migration journey, or developed in the host country during the adaption to new conditions of life. General data about the mental health of migrants reveal higher than expected levels of anxiety, depression, Post-Traumatic Stress Disorder (PTSD), panic disorder and agoraphobia [3, 4]. However, this data is rarely gender-disaggregated [5], rendering the accurate study of the mental health status of migrant women difficult.

### 3.2 Women’s Migration and Mental Health

In the migration literature, the stages of migration are described to be: 1) pre-migration; 2) migration; and 3) post-migration [6]. The last stage, post-migration, could be further divided into three sub-stages: overcompensation; decompensation; and acculturation or assimilation. The mental health of migrants seems to be influenced by experiences in the home country before migration, the process of migration itself, and the living conditions in the country of settlement [7].

In each of these stages, migrants are exposed to specific conditions, which in turn determine vulnerability to mental health problems. Many of these experiences are similar for both men and women; however, some variables are more specifically related to the female gender, because different kind and level of insecurity and violence are experienced by migrant men and women, based on social and economic conditions of home countries, of transit countries and host countries, and the relations of power between husband and wife [5].

Although migrant women face gender-related specific addicted vulnerability, there is very little data focusing at the psychological and psychopathological status of migrant women. On the other hand, there is a significantly larger amount of literature on gender-based violence and abuse, which has been documented to have psychosocial consequences.

Women are vulnerable to violence during the migration journey. Some of the violence is cross-cutting across all parts of the journey (for example, gender-based violence can occur at all points); and some of it is particular to and experienced during the different parts of the journey.

Sexual and Gender-Based Violence (SGBV) is the term generally used to define violence directed against a person on the basis of gender or sex. In the field of migration, SGBV is a recurring phenomenon, including five dimensions of violence: physical, psychological, sexual, socio-economic and cultural harmful practices, such as threats, coercion, or deprivation of freedom on the basis of a person’ sex or gender [8]. SGBV like exploitation, forced and early marriage, sexual, and domestic violence are highly prevalent during migration [9] and cause psychopathological and psychosocial effects ranging from social stigma, isolation, and psychological distress to psychiatric conditions such as Post-Traumatic Stress Disorder, Depression, Adjustment Disorders, Trauma and Stressor-related Disorders [10-15].

### 3.3 Women’s Journey and Violence

#### 3.3.1 Pre-migration

The underlying reasons for women to migrate must be looked for in their country of origin where political, cultural and religious circumstances can push them to escape [16]. In sub-saharian African countries, traditionally woman has got a subordinate position to man; she is less educated, she grows up to become a good wife and to be submissive to her husband; she has got a lower economic status and her main occupation is the management of family and domestic tasks. Often, according to gender norms and values, she is forced to marriage (usually at a very early age) and, in muslim countries [17, 18], to accept polygamous relationships, too.

In this cultural framework, she learns to see violence as a part of the marriage and as a right of husband to exercise control within marriage [19, 20]. Despite existing domestic laws prohibiting these practices, common types of gender-related persecutions are Intimate Partner Violence Against Women (IPVAW), Female Genital Mutilation (FGM), forced marriage or forced abortion or sterilization, rape and war-related violence [21],and hostage-taking’ by policy [22]. Depression, psychoticism [17, 18, 23] and trauma related disorders [24] are common outcomes of these experiences.

Although the magnitude of the issues and related results, it is difficult to have reliable data. For example, phenomenon of forced marriage is still relatively under-reported [25], even it is perpetuated by African immigrant people in hosting countries, too.

More data are available about physical and sexual violence against women. Even if SGBV is a universal reality, it appears to worsen as one goes south in the continent of Africa. Intimate Partner Violence Against Women (IPVAW) is a very common violence exercised by the husband over the woman [26] and, despite the serious consequences in terms of general health and mental health of women, it is still part of the values and norms of the South Africa people.

According to a comparative analysis of 17 sub-Saharan countries, IPVAW is targeted as a justifiable act both by men and women, particularly in case of “neglecting children”, “going out without informing husband”, “arguing back with the husband” or “refuses to have sex with her spouse”. This practice seems to be more acceptable to younger [27], less educated, poorest, living in rural areas, with less access to media women [28]. Moreover, men seem to be consistently less likely to justify wife-beating than women who consider the use of violence a way to define and maintain the difference of male and female roles [29]. The relationship between IPVAW and mental disorders has been explored in a meta-analysis: the weighted mean prevalence of mental health problems women victims of intimate partner violence was 47.6% in 18 studies of depression, 17.9% in 13 studies of suicidality, 63.8% in 11 studies of Post-traumatic Stress Disorder (PTSD), 18.5% in 10 studies of alcohol abuse, and 8.9% in four studies of drug abuse [30].

The Female Genital Mutilation/Cutting (FGM/C), or female circumcision, is another common form of violence against women in sub-Saharan African countries. It is a practice determining the removal of all or part of the external female genital in the absence of medical needs [31].

According to UNICEF data, 200 million girls and women from 30 countries mainly in Africa, the Middle East and Asia suffered this violence and 15 million girls are at risk of experiencing it by 2020 [32]. However, it is a rights violation in the context of international and national policy and can cause physical problems (infection, infertility), and mental health consequences [33].

This violent practice is perpetuated because of specific religious, cultural and traditional variables determining the female gender role, such as: girls’ virginity before marriage, monogamy in marriage, sexual availability to husband, the production of legitimate male heirs in order to insure husband’s patrilineage.Worries about marriageability and social acceptance, and the concern to loss of protection by other women and all the community if a girl refuses to suffer FGM are further reasons why this violence is still going on [34].

Studies conducted in South Africa are aimed at understanding the risk factors related to FGM. In Kenia, a study conducted on 0–14-year-old girls showed that daughters of cut women were highly likely to live the same experience. The likelihood of a girl being mutilated increased with the proportion of women who were cut, who supported FGM/C continuation, and who believed FGM/C was a religious obligation [31]. Achia [35], found a correlation between a woman’s current FGM/C status and her support for the continuation of the practice, among 15-49 years old women.

In Tanzania, it is estimated that 7.9 million women and girls have undergone FGM [36]. According to the findings of Galukande *et al*. [37], out of the 675 female study participants, 69.2% reported to have been cut at varying ages and 15.8% respondents lived it under the age of 11 years.

Others forms of violence are caused by political conditions: inthe war-torn regions, many women have been raped [25], have witnessed violent deaths and injuries, have lost families or have been victims of sexual abuse. Moreover, population displacement, damage to infrastructure, rising unemployment and erosion of welfare systems resulting from conflict can be a trigger for the development or progression of psychological distress or mental illness in women and in terms of trauma related disorders [24], depressive disorders and anxiety disorders [38].

According to the Euro-Mediterranean Human Rights Network data [39], many innocent women are arrested and become objects of physical, sexual and psychological violence; others are used as bargaining chip for hostage exchange. These traumatic events can determine the onset of psychological diseases and an increasing of internments in psychiatric hospitals [39].

All these experiences are the reasons why women can choose to leave their country, or they are part of their background, and they can be trigger for the development of additional traumatic events and for the onset of further psychological problems.

#### 3.3.2 Migration Journey

Many characteristics of the journey to the host country, including the duration of the journey, cause migrant women to be particularly vulnerable to experiencing psychological distress, violence, and other traumatic experiences. SGBV is part of the journey for most of the women [40], and considering the established link between SGBV and psychological and mental health variables, it is worthwhile examining the documented experiences of violence against migrant women that occur during the migration journey.

Women’s experiences of SGBV on the migrant journey include being forced to have sex by smugglers, being used as a bargaining chip by their family to get a seat on boats, being compelled to sell their bodies in order to buy a seat for their dependents or to avoid torture and violence, or being used as exchange goods in exchange for prostitution or other illegal services [41]. The use of small, run-down, and old boats makes the journey particularly unsafe for women and children, who are often forced to take the worst and most dangerous seats [42]. The unsafe conditions of the journey are often confirmed at landing in the new country. As a case example, 368 refugees died in 2013 when the boat that was being used sank while trying to reach the coast of the Sicilian island of Lampedusa. 50% of those who perished were women and 20% were children, and despite the rescue interventions of the Italian coast guards and fishermen only 160 people could be saved, of which only 15% were women and 5% were children. This was due to their places in the bottom of the boat which placed them at greater risk for drowning, and also because they were thrown into the sea first by smugglers because of their perceived weakness [42, 43].

In a study of women’s experiences of violence during migration, Syrian women on the Euro-Mediterranean transit region reported experiences with violence during the passage [44]. The study also noted that upon arrival at the country of destination, many Syrian women were averse to discussing their experiences with SGBV during their journey and cited cultural barriers and fears as a possible reason for under-reporting [44].

Migrants, including women, are subjected to violence at the hands of the state authorities [45]. An examination of the experiences of migrant women arriving in Kos, Greece as part of their migration journey to Europe in 2015 found that immigration and asylum policies also pose a risk factor for women [5]. Women traveling alone or without male partners “are particularly vulnerable to attack, and there were several accounts of women who had been raped and sexually assaulted on their journeys” [5]. For example, Syrian women, who began increasingly travelling alone or without a male partner, faced particular insecurities and aid workers reported that the experiences of danger on the migration journey led to levels of distress and trauma for women arriving in Kos. The reliance on smugglers on the journey subjected migrant women to exploitation, danger, harm, as well as high economic cost. Aid workers also reported that women traveling with their husbands were at risk of Intimate Partner Violence during the journey as well as upon arrival.

Women were also at risk of attacks by various police or military forces in the countries crossed on the way to Europe [5, 46]. Instances of violence, torture and detention at the hands of officials were experienced by migrants passing through Libya en route to Europe. Migrants are also vulnerable to human trafficking and smuggling industries and women in particular can be subjected to sexual violence during the journey [46].

#### 3.3.3 Post-migration

Refugees are particularly at risk for poor mental health [47, 48]. In the post-migration period, isolation and loneliness are common feelings as underlined in most studies on the mental health of refugee [49]. Social isolation is a salient factor predicting poor mental health, especially among women [50].

The post-migration period can be defined as a long-lasting process of inclusion and acceptance of migrants in the core institutions, relations and statuses of the host countries. It can require enormous resources and personal skills for migrants to activate themselves in a process in which they must quickly learn a new culture, with its rights, and try to access to positions and statuses. Social inclusion process can be very long and opposed by various difficulties such as family separation, the lack of social support, the need to learn language, and facing with discrimination and poverty. Some of those stressors (cultural, social and personal too) are main themes influencing the mental health especially of refugee women [51].

During the post-migration stage, it is also possible that migrants end up in refugee camps for months or even years, where living conditions are highly stressful and can expose them to the development of stress-related disorders, to which women seem particularly at risk of being exposed. Following exposure to a traumatic event indeed, women are approximately twice as likely as males to develop PTSD [52]. The reason can be explained with insufficient social support resources, gender-specific acute psychobiological reactions to trauma [53], or of assaultive violence [52]. According to a systematic review aimed to study violence among Asylum Seekers (AS) to high-income countries, the incidence of SGBV is high in these situations [54]. Over one third (35.7%) of women in detention centers have faced sexual harassment by detention officers, and during medical consultations the rate of sexual violence was reported by AS to be 44.2%. In addition, four studies reported sexual torture among torture victims [55]. Although there is a clear lack of data on the health of African female migrants once they have arrived in a European country, a study performed in Turin has shown that almost all the women reported to be abused in Libya, with sexual violence, one third of women had at least a scar and a lot of them reported a female genital mutilation [56].

Women often do not access treatment following the violence they have suffered not only during the journey but in some cases, even once they arrive in the host country [57].

According to a study conducted to investigate violence experience among immigrants and refugees in Italy, 56% of the women had been victims of at least one form of violence (psychological, physical, economic and sexual) during their permanence in Italy and 28.5% experienced multiple incidents of violence [58]. The reason could be that they are often deprived of social network support, they have no knowledge and awareness of how the psychological and clinical support services work in the host countries, another important barrier to access to care can be language, cultural background and social pressure [59].

MSF aid workers in Kos, Greece also reported that migrant women who experienced Intimate Partner Violence from their husbands or male partners were often unable to leave the abusive relationship due to the high level of social and financial insecurity faced in the country of destination [5].

A study performed in Spain has similarly highlighted that there could be a strong association between immigration status characteristics such as personal, social support, family among immigrant women living in Spain and Intimate Partner violence [60].

Even if the exposure to the risk of suffering further violence once arrived in the host countries for migrant women is very high, very few studies have highlighted this phenomenon [61-63].

A study performed in Pavia has shown that only half (49.7%) of the sample of migrant women receive a diagnostic procedures and/or drug prescription and only a third had a preventive intervention or chronic disease control [64]. There is a clear need to understand and implement new strategies to access and remove barriers to care concerning the model of health-care utilization among immigrants. To facilitate the access support network to national health services, a key role could be played by the third-sector organizations’ given that it is clear that public policies do not completely meet the needs [64].

Some studies have been carried out on the female population host in camps provided in border areas from which the migration process has started.

Studies from refugee camps with Tuareg refugees in Burkina Faso have compared psychological distress in male and female people. The results show higher rates were present in women; particularly over 40 years old women were at higher risk for PTSD symptoms, probably due to their awareness [13, 65].

Depression is present in terms of maternal depression among Syrian refugees in Lebanon, associated with younger age of marriage, illegal residence, increased exposure to domestic violence and a history of mental illness, more than among Lebanese mothers [66].

Several studies implementing psychosocial approaches aimed at improving mental health of migrants have been performed, both in host countries and in borders hot spot. Studies that had been supported or conducted in group setting or with the help of someone of the same communities seem to be considered more effective [67].

Some protocols involved family members or community activation of migrants in the host country and showed their effectiveness in facilitating access to care services [68-70].

Anyway, there is a clear lack of interventions designed to meet the particular care needs of migrant women in order to produce positive mental health outcomes once they arrive in the host countries. Likewise, targeted policies to promote mental health and prevent mental disorders among women migrants offering social, emotional, support to address the barriers against mental health services in Western host countries seem to be totally absent.

## 4 DISCUSSION

This narrative review shows the lack of data about psychological status of women emigrating to countries of Mediterranean area and about the presence of devoted mental health services.

Most of these women, when they arrive at the coveted countries, show wounds, signs and symptoms of violence suffered and of their psychological suffering. Unfortunately, too few studies have been conducted to collect data with the aim to have a plausible view of the experiences they lived, in the countries of origin or during the journey or at the arrival in the Mediterranean countries.

## 5 LIMITATION

To our knowledge, this is the first narrative review about mental health status of migrant women in Mediterranean area.

The main limitation that needs to be taken into account, is determined by the lack of specific data about psychological status of migrant women. No studies have been conducted to highlight specific mental health effects of migration on women, neither in native countries, during the journey and their arrival in the destination country. It has denied possibility of studying the issue and has determined the need for looking for data about physical and psychological violence experienced by women in every step of the migration journey.

## CONCLUSION

Despite migration is painful, stressful and traumatic both for males and females, there is a dearth in the literature about the psychological distress experienced by women. Our research did not yield enough results in order to be able to answer to our over-arching question about the psychological health of female migrants. We believe that further research is needed in order to better understand gender-specific variables for mental health during migration, including violence against woman, so as to be able to apply good practices of care and prevention.

The existing data about Sexual and Gender-Based Violence during migration underlines the need for a different set of policies and care approach specifically aimed towards helping women not only manage their psychological and trauma-related disorders but also towards improving their resilience in the face of these variables [71]. Improving accommodation and living conditions, delivering mental health assistance through primary healthcare, creating community-based multidisciplinary mental health teams [72], using simultaneous translation to break down language and communication barriers, and training primary helpers and stakeholders in basic elements of psychology and intercultural competence are essential for delivering services to be utilised by migrant women.

We agree with the Editorial of *The Lancet Public Health* [73], which states that “protecting migrants during and after migration is our responsibility”. European public health advocates cannot ignore the needs, pain and vulnerability of migrant people. The role of academics and research now is highlighting the issue, initiating the discussion, and promoting new approaches based on growing evidence and literature. None can pull out.

Our idea is that all the stakeholders - at cultural, educational, national and international policy level - shouldfeel involved in this issue. An implementation of care system for women directed towards primary prevention of violence and psychological distress (in the home countries, especially), and secondary and tertiary prevention in the countries involved in the migration journey is urgent.

It is necessary to create a systematic approach, including cultural, social, political, educational and health perspectives, to give an answer to the psychological suffering of migrant women.
